# Human Germinal Center CD4^+^ CD57^+^  T Cells Act Differently On B Cells Than Do Classical T-Helper Cells

**DOI:** 10.1155/1995/76790

**Published:** 1995

**Authors:** Farida Bouzahzah, Alain Bosseloir, Ernst Heinen, Léon J. Simar

**Affiliations:** 1 Institute of Human Histology University of Liege rue de Pitteurs 20 Liege B-4020 Belgium; 2 Institute of Human Histology rue de Pitteurs Liege 20 B-4020 Belgium

**Keywords:** Germinal center, T-helper cells, CD57+ T cells, cell proliferation

## Abstract

We have isolated two subtypes of helper T cells from human tonsils: CD4^+^ CD57^+^ cells, mostly located in the germinal center (GC), and CD4^+^ CD57^-^ cells, distributed through the interfollicular areas but also present in the GC. In a functional study, we have compared the capacities of these T-cell subtypes to stimulate B cells in cocultures. In order to block T-cell proliferation while maintaining their activation level, we pretreated isolated T cells with mitomycin C prior to culture in the presence of B cells and added polyclonal activators such as PHA and Con A, combined or not with IL-2. Contrary to CD4^+^ CD57^-^ cells, CD4^+^ CD57^+^ cells did not markedly enhance B-cell proliferation. Even when sIgD^-^B cells typical of germinal center cells were tested, the CD4 CD57 cells had no significant effect. This is in accordance with the location of these cells: They mainly occupy the light zones of the GC where few B cells divide. Even when added to preactivated, actively proliferating cells, CD4^+^ CD57^+^ cells failed to modulate B-cell multiplication. On the supernatants of B-cell-T-cell cocultures, we examined by the ELISA technique the effect of T cells on Ig synthesis. Contrary to CD57^-^ T cells, whose effect was strong, CD57^+^ T cells weakly stimulated Ig synthesis. More IgM than IgG was generally found. Because CD57 antigen is a typical marker of natural killer cells, we tested the cytolytic activity of tonsillar CD4^+^ CD57^+^ cells on K562 target cells. Unlike NK cells, neither CD4^+^CD57^+^ nor CD4^+^ CD57^-^ cells exhibit any cytotoxicity. Thus, germinal center CD4^+^ CD57^+^ cells are not cytolytic and do not strongly stimulate either B-cell proliferation or Ig secretion. CD4^+^ CD57^-^ cells, however, enhance B-cell proliferation and differentiation, thus acting like the classical helper cells of the T-dependent areas.


*Developmental Immunology*, 1995, Vol 4, pp. 189–197
Reprints available directly from the publisher
Photocopying permitted by license only

© 1995 Harwood Academic Publishers GmbH
Printed in Singapore

### Human Germinal Center CD4$^+$CD57$^+$ T Cells Act Differently On B Cells Than Do Classical T-Helper Cells

FARIDA BOUZAHZAH, ALAIN BOSSELOIR, ERNST HEINEN,* and LÉON J. SIMAR

*Institute of Human Histology, University of Liège, rue de Pitteurs, 20, B-4020 Liège, Belgium*

We have isolated two subtypes of helper T cells from human tonsils: CD4$^+$CD57$^+$ cells, mostly located in the germinal center (GC), and CD4$^+$CD57$^-$ cells, distributed through the interfollicular areas but also present in the GC. In a functional study, we have compared the capacities of these T-cell subtypes to stimulate B cells in cocultures. In order to block T-cell proliferation while maintaining their activation level, we pretreated isolated T cells with mitomycin C prior to culture in the presence of B cells and added polyclonal activators such as PHA and Con A, combined or not with IL-2. Contrary to CD4$^+$CD57$^-$ cells, CD4$^+$CD57$^+$ cells did not markedly enhance B-cell proliferation. Even when sIgD$^-$B cells typical of germinal center cells were tested, the CD4$^+$CD57$^+$ cells had no significant effect. This is in accordance with the location of these cells: They mainly occupy the light zones of the GC where few B cells divide. Even when added to preactivated, actively proliferating cells, CD4$^+$CD57$^+$ cells failed to modulate B-cell multiplication. On the supernatants of B-cell–T-cell cocultures, we examined by the ELISA technique the effect of T cells on Ig synthesis. Contrary to CD57$^-$ T cells, whose effect was strong, CD57$^+$ T cells weakly stimulated Ig synthesis. More IgM than IgG was generally found. Because CD57 antigen is a typical marker of natural killer cells, we tested the cytolytic activity of tonsillar CD4$^+$CD57$^+$ cells on K562 target cells. Unlike NK cells, neither CD4$^+$CD57$^+$ nor CD4$^+$CD57$^-$ cells exhibit any cytotoxicity. Thus, germinal center CD4$^+$CD57$^+$ cells are not cytolytic and do not strongly stimulate either B-cell proliferation or Ig secretion. CD4$^+$CD57$^-$ cells, however, enhance B-cell proliferation and differentiation, thus acting like the classical helper cells of the T-dependent areas.

KEYWORDS: Germinal center, T-helper cells, CD57$^+$ T cells, cell proliferation.

### INTRODUCTION

T lymphocytes purified from human tonsils are mainly CD4$^+$ cells (Sugiyama et al., 1976, 1984, 1987; Plum et al., 1987), CD8$^+$ T cells being proportionally less numerous in tonsils than in the peripheral blood (Sugiyama et al., 1987). The prevalence of CD4$^+$ cells in tonsils suggests that tonsillar T lymphocytes are functionally oriented to helper activity (Sugiyama et al., 1987). Several immunohistochemical studies have revealed the existence of tonsillar T cells expressing the CD57 antigen characteristic of cells with NK activity (Okada et al., 1988). These cells are essentially located in the GC light zone; a few are also found in the interfollicular T-dependent areas and the mantle zones (Bouzahzah et al., 1993). In a previous ultrastrutural morphological study, we have shown that these cells are medium-sized and do not contain the large granules typical of blood NK cells (Bouzahzah et al., 1993). Phenotypically, these cells do not express Leu 8, CD16, or CD11b, so they clearly differ from classical NK cells (Si and Whiteside, 1983; Verlardi et al., 1985, 1986a). These CD4$^+$CD57$^+$ cells do not express CD25 (IL-2 receptor), CD71 (transferrin receptor), or HLA-DR, but do express CD69 (Bowen et al., 1991). Thus, they should be viewed as preactivated rather than fully activated cells. Their ability to produce cytokines is much debated (Velardi et al., 1986b; Bosseloir et al., 1989, 1991; Bowen et al., 1991; Butch 1993).

There is no available data as to the function of CD4$^+$CD57$^+$cells. In this report, we compare the effects of CD4$^+$CD57$^+$ and CD4$^+$CD57$^-$ cells on

* Corresponding author: E. Heinen, Institute of Human Histology rue de Pitteurs, 20 B-4020 Liège, Belgium. Telephone number: 32-41-66 51 74 Fax number: 32-41-66 51 73.

B-cell proliferation and differentiation in Ig-secreting cells. Using several procedures (nylon wool columns, and selections by dynabeads and MACS), we have purified both populations of T-helper cells and cocultured them with B cells isolated from tonsils. We have compared the capacity of B cells to incorporate tritiated thymidine or to produce IgM or IgG in these two types of coculture. We pretreated the T-cell populations with mitomycin C to prevent their multiplication but added various activators to the cultures. In another series of experiments, we tested the cytotoxicity of $CD4^+CD57^-$ and $CD4^+CD57^+$ in the presence of K562 target cells.

### RESULTS

### Effects of $CD57^+$ and $CD57^-$ T Cells on B-cell Proliferation

To test the effects of $CD57^+$ and $CD57^-$ cells on B-cell proliferation, cell populations enriched in these cells were added to highly purified B cells. The purity of the T-cell populations used, as estimated by flow cytometry, exceeded 94% ($CD57^+$ cells) or 96% ($CD57^-$ cells). Before being cocultured, the T-cell populations were treated with mitomycin C to minimize their capacity to incorporate [$^3$H]-thymidine. The cells were stimulated, however, with PHA (2 μg/ml) to keep them activated. As a control, B cells were cultured alone. Little or no proliferative activity was observed in B-cell populations cultured alone or in mitomycin-C-treated T cells. Coculturing with unsorted $CD4^+$ T cells or with purified $CD4^+CD57^-$ cells markedly increased B-cell proliferation (Fig. 1). $CD57^+$ cells had only a very slight enhancing effect on B-cell proliferation. Next, we reasoned that germinal center B cells might react differently than the total B-cell population. Therefore, after eliminating $IgD^+$ cells from the total tonsillar B-cell population, we cocultured either the total B-cell population or the sorted $IgD^-$ B cells with mitomycin-C-treated T cells in the presence of PHA (Fig. 2). The results obtained were similar to those of the previous experiments: the $CD57^+$T cells exerted only a weak enhancing effect on B or $IgD^-$ cell proliferation in comparison to the marked effect of $CD57^-$ T cells.

The previous experiments showed that $CD57^+$ T cells only slightly increase the proliferative activity of B cells. Considering that these $CD57^+$ cells might not be sensitive to PHA, we performed a new series of experiments in which mitomycin-C-treated $CD57^+$ or $CD57^-$ cells were cocultured with B cells in the presence of PHA or ConA, combined or not with IL-2. B-cell proliferation was measured after 48 hours (Fig. 3). Addition of IL-2 to PHA only slightly improved the uptake of tritiated thymidine by B cells cocultured with the $CD57^+$ cells. A much better response was measured when these cells were stimulated with ConA and IL-2. Similar results were obtained in cocultures of B and $CD57^-$T cells, but the level of proliferation was greater than in cultures composed of B and $CD57^+$ cells. None of the activators used (ConA, PHA, IL-2) elicited any significant proliferative response in B cells cultured without T cells.

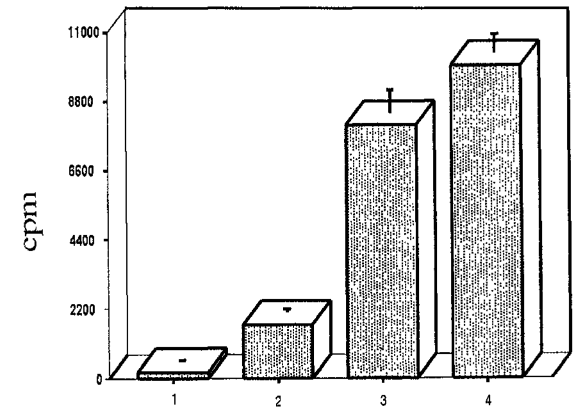

FIGURE 1. Proliferation of B cells in presence of $CD57^+$ or $CD57^-$ cells. B cells ($10^5$ cells per well) were incubated with the $CD57^+$ or $CD57^-$ cells ($3 \times 10^5$ cells/well) and examined for proliferation after 48 hours of culture. The $CD57^+$ and $CD57^-$ cells were pretreated with mitomycin C (50 μg/ml for 45 min) and activated with PHA (2 μg/ml). Levels of tritiated thymidine uptake are indicated in CPM. The results are expressed as the mean of quadriplicate cultures and are representative of 4 experiments performed. In 1: B cells alone; 2: B cells and $CD57^+$ cells; 3: B cells and $CD57^-$ cells; 4: B cells and unsorted $CD4^+$ cells.

### Effects of $CD57^+$ and $CD57^-$ Cells on the Proliferative Activity of Preactivated B Cells

The previous experiments were performed with freshly isolated B and T cells. To see whether the T-cell population might inhibit active B-cell proliferation, the B cells were preactivated for 24 hours with SAC or with anti-CD40 antibody combined with anti-Ig (respectively at 100 ng/ml and 1/600). The sorted T cells were treated with mitomycin C before coculturing with preactivated B cells. PHA was included throughout the culture period.

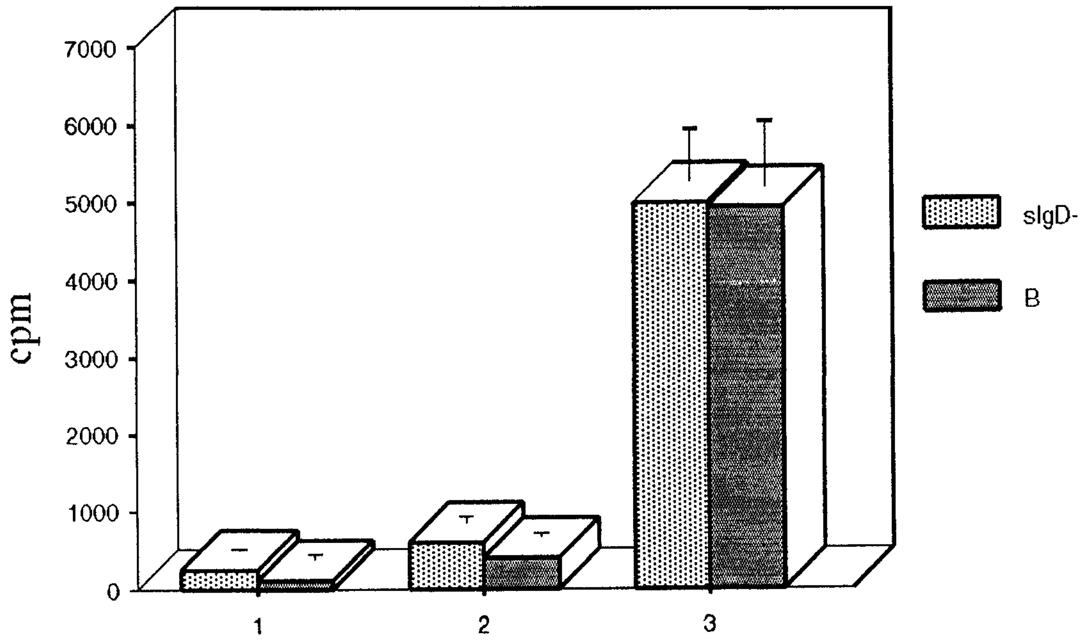

FIGURE 2. Proliferation of unsorted B cells or IgD$^-$B cells in presence of CD57$^+$ or CD57$^-$ cells. B cells or sIgD$^-$B cells ($10^5$ cell per well) were cocultured with mitomycin-C-treated CD57$^+$ or CD57$^-$ cells (3 x $10^5$ g/well). sIgD$^-$ cells were sorted using anti-IgD$^-$ mAb and the MACS procedure. PHA was added whole during culture duration to maintain T-cell activation. B-cell proliferation was measured after 48 hours. The results are expressed as the mean of quadriplicate cultures. In 1: B cells and sIgD cells alone; 2: B cells and sIgD$^-$ cells and CD57$^+$; 3: B cells and sIgD$^-$ cells and CD57$^-$.

As shown in Fig. 4, the proliferation of B cells alone was markedly increased in the presence of all the different activators. Addition of CD57$^+$ cells increased the proliferative response when the B cells were stimulated with SAC, but not when they were preactivated with anti-Ig and anti-CD40. Addition of CD57$^-$ T cells was always followed by marked stimulation of the preactived B cells.

### Effects of CD57$^+$ or CD57$^-$ T Cells on Ig Secretion

Tonsillar B cells were cultured alone or in the presence of mitomycin treated CD57$^+$ or CD57$^-$ T cells. The ratio of T to B cells was 3/1. PHA or ConA, combined or not with IL-2, was added as a stimulator to the culture medium. The supernatants

TABLE 1
Effect of CD57$^+$ or CD57$^-$ Cells on Ig Production

| Addition to Culture Medium | Secreted Ig (ng/ml) IgM | IgG |
|---|---|---|
| B cells + PHA + IL2 | 600 ± 48 | 994 ± 180 |
| B cells + ConA + IL2 | 600 ± 48 | < 733 |
| B cells alone | 600 ± 48 | 760 ± 225 |
| B cells + CD57$^+$ cells + PHA | 600 ± 48 | 922 ± 180 |
| B cells + CD57$^+$ cells + PHA + IL2 | 3,216 ± 211 | 1,899 ± 225 |
| B cells + CD57$^+$ cells + ConA | 2,544 ± 192 | 594 ± 57,6 |
| B cells + CD57$^+$ cells + ConA + IL2 | 3,609 ± 240 | 877 ± 180 |
| B cells + CD57$^-$ cells + PHA | 3,580 ± 259 | 3,271 ± 495 |
| B cells + CD57$^-$ cells + PHA + IL2 | 10,170 ± 1,350 | 5,566 ± 644 |
| B cells + CD57$^-$ cells + ConA | 3,926 ± 384 | < 733 |
| B cells + CD57$^-$ cells + ConA + IL2 | 11,475 ± 1,485 | 733 ± 180 |

Purified tonsillar B cells ( 1 x $10^5$) were cocultured with mitomycin-C-treated CD57$^+$ or CD57$^-$ cells (3 x $10^5$) in 96-well plates in a total volume of 200 μl per well in presence of different combinations of activators (PHA; 2 μg/ml; ConA: 10 μg/ml; IL-2: 10 U/ml). After 10 days, the culture supernatants were harvested and analyzed for the presence of IgG and IgM by specific ELISA. The results are representative of 3 experiments.

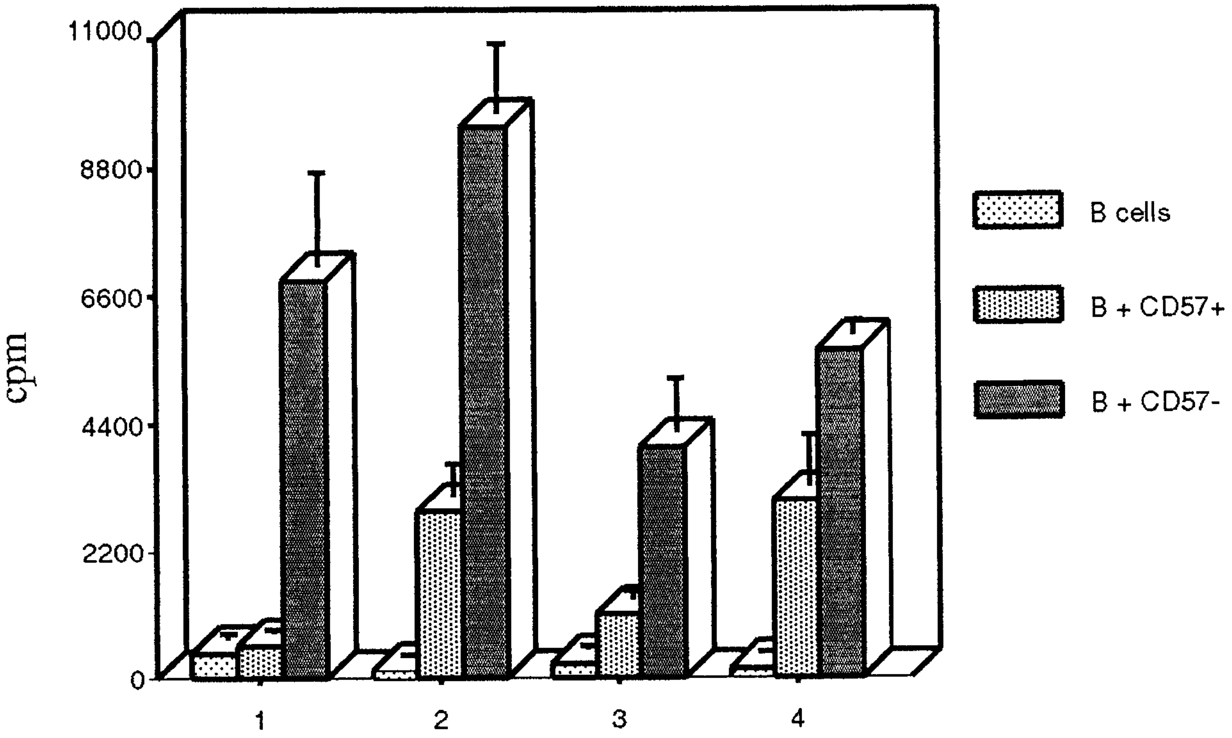

FIGURE 3. Proliferation of B cells in presence of CD57⁺ or CD57⁻ cells and different activators. B cells ($10^5$ cells/well) were cocultured with mitomycin-C-treated T cells (3 × $10^5$ cells/well). Tritiated thymidin incorporation was measured after 48 hours. No or very low proliferative activity was observed in B cells cultured alone or in mitomycin-C-treated T-cell populations. T cells ($CD57^+$ or $CD57^-$) were activated with PHA (2 μg/ml) or Con A (10 μg/ml) combined or not to IL-2 (10 U/ml): 1: PHA; 2: PHA and IL-2; 3: ConA; 4: ConA and IL-2. These results are representative of 3 experiments performed and are expressed as the mean of quadriplicate cultures.

were collected after 10 days of culture and their IgM and IgG contents analyzed by ELISA. The results are presented in Table 1, B cells cultured alone did not produce large amounts of Ig. Addition of T cells generally increased Ig synthesis, $CD57^-$ T cells being much more effective than $CD57^+$ T cells. IgM were secreted in larger quantities than IgG, especially when IL-2 was present in the medium. ConA was a better inducer of IgM production than PHA, but less effective than PHA at increasing IgG secretion. Thus, IgM secretion was highest when B cells were cocultured with $CD57^-$ T cells stimulated by ConA and IL-2, whereas IgG secretion was highest in B cells cocultured with $CD57^-$ cells stimulated with PHA and IL-2.

### Cytolytic Activity of $CD57^+$ and $CD57^-$ T Cells

Neither freshly isolated nor preactivated $CD57^+$ or $CD57^-$ cells exerted any cytolytic activity against K562 target cells. Comparatively high cytolytic activity was measured in the positive controls: for example, 60% $^{51}Cr$ release at 20:1 and 50% at 10:1 (effector: target ratio).

### DISCUSSION

Helper activity toward B lymphocytes can efficiently be induced *in vitro* by stimulation of human T cells with various mitogens (Hirohata et al., 1988; Patel et al., 1993; Dembech et al., 1992). In this report, we examine the effect of $CD4^+CD57^+$ and $CD4^+CD57^-$ tonsillar T-cell populations on the proliferation and terminal differentiation of B cells isolated from tonsils.

The $CD4^+CD57^-$ cells are representative of the T-cell populations of the interfollicular areas and GC, whereas the $CD4^+CD57^+$ are essentially GC T cells. Although the CD57 Ag is also expressed by NK cells, the tonsillar $CD57^+$ cells are morpholog-

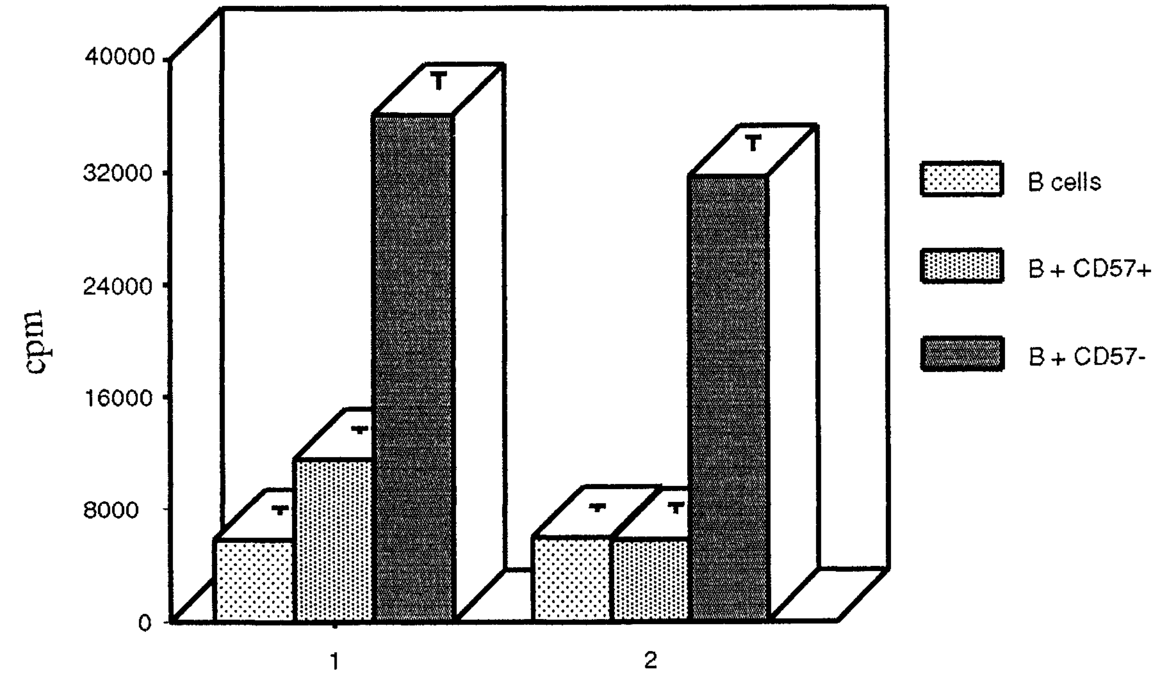

FIGURE 4. Effect of $CD57^+$ or $CD57^-$ cells on the proliferation of preactivated B cell. B cells ($10^5$ cells) were preactivated with: 1:SAC (at a final dilution of 1/10,000) or 2: anti-Ig combined to anti-CD40 (respectively at 1/600 and 100 ng/ml). After 24 hours of culture. mitomycin-C-treated $CD57^-$ or $CD57^+$ cells (3 x $10^5$ cells/well) were added in presence of PHA (2 μg/ml). Thymidin incorporation by B cells was measured after 48 hours. Results are expressed as the mean of quadriplicate cultures and are representative of 5 experiments.

ically and functionally different. We have, in fact, evaluated the cytolytic activity of these cells by the $^{51}Cr$ release method, using human K562 cell as targets. Unlike classical NK cells, tonsillar $CD4^+CD57^+$ manifest no NK activity, even when preactivated with PHA for 24 hours. Our results agree with those of Velardi et al. (1986a, 1986b). Thus, expression of this NK-related antigen does not reflect a cell's cytolytic potential, but characterizes a subset of $CD4^+$ T-helper cells in germinal centers.

When $CD57^-$ or $CD57^+$ cells were cocultured with B cells, B-cell proliferation was slightly increased by the $CD57^+$ cells but enhanced thirty-fold in the presence of $CD57^-$ cells or unsorted $CD4^+$ cells. This finding is in line with results reported by Velardi et al. (1986b) showing that $CD57^+$ cells do not produce B-cell growth factors. Several studies have indicated that a subpopulation of germinal center B cells can proliferate in response to low concentrations of IL-2 (Forman and Pure, 1991). In our experiments, $CD57^+$ T cells did favor B-cell proliferation when IL-2 was added to the medium. IL-2 by itself was not sufficient to stimulate B-cell proliferation, because B cells cultured alone in the presence of IL-2, combined or not with PHA or ConA, failed to proliferate. IL-2 appears to act on T cells, which then stimulate B cells. This activation requires also direct T-cell–B-cell interaction (Clark and Lane, 1991; Tohma and Lipsky, 1991; Liu et al., 1992; MacLennan et al., 1992; Moller, 1992) because physical separation of the two cell types with a semipermeable membrane precludes B-cell activation. Cytokines, particularly IL-2 (Porwit-Ksiazeck et al., 1983) and IL-4 (Fliedner et al., 1964; Defrance et al., 1988), appear to play an important role. The capacity of $CD57^+$ cells to produce IL-2, IL-4, and other cytokines is much debated: Bosseloir et al., (1989, 1991) using *in situ* hybridization failed to detect any IL-6 or IL-4-secreting cells in tonsil germinal centers, whereas Butch et al. (1993) say they have detected IL-4 mRNA in GC T cells. Currently, we are checking the presence of cytokines in supernatants of $CD57^+$ cell cultures and analyzing mRNA contents of freshly prepared $CD57^+$ T cells.

At this level of our work, we can conclude that $CD57^+$ T cells, contrary to $CD57^-$ T cells, whose effect is strong, exert a very weak effect on B-cell proliferation, even on IgD$^-$ B cells arising in part in the germinal centers. This conclusion is in agreement with immunohistological studies showig that CD57$^+$ T cells are predominantly located in the germinal center light zone. One should remember that B-cell proliferation occurs mainly in the dark zone (O'Garra et al., 1988; Freedman et al., 1992), where few CD57$^+$ T cells are found. It has been suggested that in the light zone, differentiation of B cells into memory cells or plasma cell precursors is accompanied by decreased B-cell proliferation (Noëelle and Snow, 1992). Because CD4$^+$CD57$^+$ cells are numerous in the light zone, we reasoned that they might play a role in curbing the proliferative activity of dividing B cells coming from the dark zone. We tested this by coculturing the two T-cell populations with B cells preactivated for 24 hours with SAC or anti-CD40 combined with anti-Ig. The CD57$^-$ T cells were found to markedly enhance B-cell proliferation whatever the stimulator used; CD57$^+$ cells, on the contrary, only slightly increased B-cell proliferation in the presence of SAC and had no effect on B cells preactivated with anti-Ig and anti-CD40.

In another series of experiments, we have shown CD57$^-$ T cells to be much more effective than CD57$^+$ cells in inducing B cells to secrete IgM or IgG. This action of T cells on Ig secretion was largely dependent upon the mode of stimulation applied. ConA, for instance, was a better inducer of IgM synthesis than PHA, whereas Ig production was highest in the presence of IL-2. B cells cultured alone exhibited only minimal Ig secretion, even in the presence of exogenous IL-2 combined or not with ConA or PHA. In fact, Dembech et al. (1992) report having failed to induce B cells to secrete significant amounts of Ig by adding various recombinant cytokines, such as IL-2, IL-4, IL-6, IFN$\alpha$, all involved in the proliferation and differentiation of B cells (O'Garra et al., 1988). Thus, optimal differentiation of human B cells appears to require not only cytokines, but also close contact with T cells (Reidel et al., 1988; Whalen et al., 1988; Noëlle and Snow, 1992). One of the molecules involved in T-cell–B-cell contact is CD40. Expression of CD40 ligand apparently occurs in activated T cells, but expression of this molecule by GC T cells (CD4$^+$CD57$^+$) remains controversial (Spriggs et al., 1993; Van den Eertwegh et al., 1993).

We have thus defined two populations of tonsillar CD4$^+$ cells that differently affect B-cell proliferation and Ig production. The functional differences between the CD4$^+$CD57$^+$ and CD4$^+$CD57$^-$ populations may be due to the ability of these cells to respond to the mitogens used. We are currently continuing to test the proliferative capacity and the expression of activation markers by these cells in response to different mitogens and accessory cells.

Further studies must be conducted to investigate the function of CD57$^+$ T cells, notably in cocultures where follicular dendritic cells are present as well as centrocytes. Indeed, CD57$^+$ cells in the light zone are adjacent to centrocytes that appear as intermediates between activated centroblasts (dark zone) and quiescent B memory cells (recirculing lymphocytes). We hypothesize that CD57$^+$ T cells may contribute to this maturation of centrocytes into B memory lymphocytes.

### MATERIAL AND METHODS

### Reagents

Collagenase, dispase, and deoxyribonuclease were obtained from Boehringer Mannheim and used at 0.005%. Magnetic beads and dynabeads coated with anti-mouse IgG were purchased respectively from Miltenyi Biotec (Germany) and Dynal (Norway). Mitomycin C was purchased from SIGMA and used at 50 μg/ml. Phytohemagglutinin (PHA, 2 μg/ml), concanavalin A (ConA, 10 μg/ml), and purified recombinant IL-2 (10 U/ml) were purchased from Boehringer Mannheim (Germany). Formalinized particles of Staphylococcus aureus strain Cowan I (SAC) were purchased as pansorbin from Calbiochem-Behring Corporation (La Jolla, CA) and used at the final dilution of 1/10,000. Nylon wool was kindly provided by Dr. Thierry Defrance (Institut Pasteur de Lyon, France).

### Monoclonal Antibodies

PE-conjugated and unconjugated anti-CD19 and anti-CD8, PE-conjugated anti-CD2 and anti-CD3 mAbs were purchased from DAKO. FITC-conjugated streptavidin was purchased from Boehringer Mannheim and used at the final dilution of 1/40. Biotin-conjugated anti-CD57 mAb was obtained from Becton Dickinson. Culture supernatants of OKT3 (anti-CD3 mAb) and OKT 11 (anti-CD2 mAb) hybridoma cell lines and the anti-CD40 mAb were kindly provided by Dr. E. A. Clark (University of Washington, Scolt, WA). Rabbit anti-human immunoglobulin coupled to polyacrylamide beads was purchased from Biorad Laboratories (Richmond, CA) and used at the final dilution of 1/600. Anti-IgD m Ab (Amersham) was used at the final dilution of 1/300.

### Isolation Procedure of Helper T Cells

Tonsils from 4- to 1-year-old children were surgically dissected and transported to the laboratory at 4°C in a physiological solution containing 0.4% bovine serum albumin (BSA). The tonsils were then cut into fragments and treated first with collagenase alone, then with a mixture of collagenase, dispase, and deoxyribonuclease.

T cells were separated from other cells by running the lymphocyte suspensions through nylon wool columns prepared by packing 50-ml syringes with nylon wool. Before the cells were added ($10^9$ cells per column), the columns were washed at 37°C with RPMI 1640 supplemented with 10% FCS. After the cells were added, the columns were eluted with RPMI–10% FCS until the cells had run two-thirds of the way through them. After incubation for 1 hour at 37°C, the columns were slowly rinsed with the eluent (RPMI–10% FCS at 37° C) and the nonadherent T cells recovered. The recovered T cells were labeled with anti-CD8 and anti-CD19 mAbs and subsequently incubated with anti-mouse IgG-coated magnetic beads. The B cells and $CD8^+$ cells were removed by applying a magnetic field for 10 min. The purity of the $CD4^+$ T-cell population exceeded 98%, as estimated by flow cytometry. An anti-CD57 mAb and a magnetic cell sorter (Becton Dickinson) were used to sort the $CD4^+$ T cells into $CD4^+CD57^+$ and $CD4^+CD57^-$ cells. Briefly, the $CD4^+$ T cells were incubated with biotin-conjugated anti-CD57 mAb for 30 min, rinsed, and placed in contact with FITC-conjugated sterptavidin for 10 min. Then they were incubated with biotinylated magnetic beads before transfer to the separation column installed in the magnetic field of the MACS apparatus. The $CD57^+$ cells were recovered by rinsing the column with a physiological solution at 4°C outside the magnetic field. As estimated by flow cytometry, the purity of the $CD57^+$ and the $CD57^-$ cells obtained by this procedure exceeded 94 and 96%. These T cells were treated with mitomycin C at 37°C for 45 min, rinsed thrice, then cocultured with B cells in the presence of PHA or ConA, combined or not with IL-2.

### B-Cell Separation

After enzymatic digestion, tonsillar mononuclear cells were incubated with 2-aminoethylioso-thiouronium-bromide-treated SRBC. Nonrosetting cells were labeled with anti-T-cell mAbs (OKT3, OKT11) and dynabeads to remove contaminant T cells. As estimated by flow cytometry with PE-conjugated mAbs (anti-CD19, anti-CD3, anti-CD2), the purity of the B-cell populations thus obtained exceeded 95%. Anti-IgD mAb and MACS were used to isolate sIgD–B cells; the purity of the $sIgD^-$ B cells, as estimated by flow cytometry, was 80%. All purification steps (rosetting, MACS) were performed at 4°C.

### Cultures

Cultures were grown in RPMI 1640 supplemented with 10% FCS, 2 mM L-glutamine, 100 U/ml penicillin, and 100 μg/ml streptomycin.

$1 \times 10^5$ B cell swere cocultured with $3 \times 10^5$ T cells per well in 96-well microtiter plates in a final volume of 150 μl (proliferation tests) or 200 μl (differentiation tests). The T cells were treated before hand with mitomycin C (50 μg/ml) at 37°C for 45 min. Different polyclonal T-cell activators (PHA, ConA, IL-2) were added to the cultures. To test the capacity of T cells to suppress B-cell proliferation, we preincubated the B cells with SAC or anti-CD40 combined with anti-Ig for 24 hours prior to addition of T cells.

The cultures were incubated at 37°C in a 5% $CO_2$ enriched atmosphere for 48 hours. A B-cell proliferation was tested by pulsing the cells with [$^3$H]thymidine (1 μCi per well) during an additional 16-hour incubation. Results were expressed as means of triplicate or quadruplicate cultures. To measure Ig production, we maintained the cultures for 10 days and analyzed the supernatants by the ELISA technique as described by Defrance et al. (1988).

### Cytolytic Test

The cytolytic activity of $CD57^+$ and $CD57^-$ T cells was tested by measuring $^{51}$Cr release from human K562 cells as described by Parker and Martz (1980). Cytolytic activity was assayed on freshly isolated $CD57^+$ and $CD57^-$ cells or on ones pre-activated for 24 hours with PHA.

Activated peripheral blood lymphocytes (PBL) were used as positive controls.

### ACKNOWLEDGMENTS

We are grateful to Elisabeth Franzen (Pathology, University of Liège) for performing the cytolytic tests, to Dr. Daele (Citadelle's Hospital), and Dr. Boniver (Bruyères Hospital) for delivering tonsils. The authors thank Ms. Jackers and Greimers for excellent technical assistance. This work was supported by the Fonds de la Recherche Scientifique Médicale.

*(Received September 27, 1994)*

*(Accepted December 23, 1994)*

### REFERENCES

Bosseloir A., Hooghe-Peters E., Heinen E., Cormann N., Kinet-Denoel C., Van Haelst L., and Simar L.J. (1989). Localization of interleukin-6 mRNA in human tonsils by in situ hybridization. Euro. J. Immunol. **19**: 2379–2382.

Bosseloir A., Hooghe-Peters E., Heinen E., Marcoty C., Cormann N., Kinet-Denoel C., and Simar L.J. (1991). Lymphatic tissues and in vivo immune response. *In* Localization of interleukin-4 and interleukin-6 messenger ribonucleic acid in human tonsils by ISH, Imhof Berrih, Ezine, Ed., pp. 315–319.

Bouzahzah F., Heinen E., Antoine N., Bosseloir A., Mancini I., and Simar L.J. (1993) Ultrastructure of CD57$^+$ cells isolated from human tonsils and blood. Eur. J. Morph. **31**: 82–86.

Bowen M.B., Butch A.W., Parvin C.A., Levine A., and Nahm M. H. (1991). Germinal center T cells are distinct helper-inducer T cells. Human Immunol. **31**: 67–75.

Butch A.W., Chung G.H., Hoffmann J.W., and Nahm M.H. (1993). Cytokine expression by germinal center cells. J. Immunol. **150**: 39–47.

Clark E.A., and Lane P.J.L.A. (1991). Regulation of human B cell activation and adhesion. Rev. Immunol. **9**: 97–127.

Defrance T., Vanbervliet B., Pene J., and Banchereau J. (1988). Human recombinant IL-4 induces activated B lymphocytes to produce IgG and IgM. J. Immunol. **141**: 2000–2005.

Dembech C., Quinti I., Cimignoli E., Albi N., Terenzi A., Gerli R., Galandrini R., Grignani F., and Velardi A. (1992). Human T-helper clones IgG production is a subclass-specific fashion. Cell. Immunol. **139**: 306–317.

Fliedner T., Kress M., Cronkite E., and Robertson J. (1964). Cell proliferation in germinal centers of the rat spleen. Ann. NY Acad. Sci. **113**: 578–594.

Forman M.S., and Pure E. (1991). T-independent and T-dependent B lymphoblasts: Helper T cells prime for interleukin-2 induced growth and secretion of immunoglobulins that utilize downstream heavy chains. J. Exp. Med. **173**: 687–697.

Freedman A.S., Munro J.M., Rhynhart K., Schow P., Daley J., Lee N., Svahn J., Eliseo L., and Nadler L. M. (1992). Follicular dendritic cells inhibit human B-lymphocyte proliferation. Blood **80**: 1284–1288.

Hirohata S., Jelinek D.F., and Lipsky P.E. (1988). T cell-dependent activation of B cell proliferation by immobilized monoclonal antibodies to CD3. J. Immunol. **140**: 3726–3744.

Liu Y.J., Johnson G.D., Gordon J., and MacLennan I.C.M. (1992). Germinal centres in T-cell-dependent antibody responses. Immunol. Today **13**: 17–21.

MacLennan I.C.M., Liu Y.J., and Johnson G.D. (1992). Maturation and dispersal of B-cell clones during T cell-dependent antibody responses. Immunol. Rev. **126**: 143–161.

Moller G. (1992). Germinal centers in the immune response. Immunol. Rev. **126**: 5–178.

Nöelle R.J., McCann J., Marshall L., and Bartlett W.C. (1989). Cognate interactions between helper T cells and B cells. III. Contact-dependent, lymphokine-independent induction of B cell cycle entry by activated helper T cells. J. Immunol. **143**: 1807–1814.

Nöelle R., and Snow E.C. (1992). T helper cells. Curr. Opin. Immunol. **4**: 333–337.

O'Garra A., Umland S., Defrance T., and Christiansen J. (1988). B cell factors are pleiotropic. Immunol. Today **9**: 45.

Okada T., Nishimua T., Yagisawa M., and Naito K. (1988). The role of natural killer cells in human tonsillar tissue focussing on the change of tonsillar time with aging. Acta Otolaryngol. (Stockholm) Suppl. **454**: 96–107.

Parker W.L., and Martz E. (1980). Lectin-induced nonlethal adhesions between cytolytic T lymphocytes and antigenically unrecognizable tumor cells and nonspecific "triggering" of cytolysis. J. Immunol. **124**: 25–35.

Patel S.S., Thiele D.L., and Lipsky P.E. (1993). Stimulus-dependent modulation of human B cell function by T cell clones. Cell. Immunol. **146**: 362–381.

Plum J., Van Cauenberge P., and De Smedt M. (1987). Analysis of activation markers on tonsillar T lymphocytes. Acta Otolaryngol. (Stockholm) Suppl. **454**: 113–117.

Porwit-Ksiazeck A., Ksiazeck T., and Biberfeld P. (1983). Leu7$^+$ (HNK-1$^+$) cells. I. Selective compartmentalization of Leu7$^+$ cells with different immunophenotypes in lymphatic tissues and blood. Scand. J. Immunol. **18**: 485–493.

Reidel C., Owens T., and Nossal G.J.V. (1988). A significant proportion of normal resting B cells are induced to secrete immunoglobulin through contact with anti-receptor antibody-activated helper T cells in clonal cultures. Eur. J. Immunol. **18**: 403–408.

Si L., and Whiteside T.L. (1983). Tissue distribution of human NK cells studied with anti-Leu7 monoclonal antibodys. J. Immunol. **130**: 2149–2155.

Spriggs M.K., Fanslow W.C., Armitage R.J., and Belmont J. (1993). The biology of the ligand for CD40. J. Clin. Immunol. **13**: 373–380.

Sugiyama M., Yamamoto K., Kinoshita Y., and Kimura S. (1976). Studies on differences in cellular immune response between the human tonsil and blood lymphocytes. Acta Otolaryngol. (Stockholm) **82**: 440–450.

Sugiyama M., Sakashita T., Cho J.S., and Nakai Yo (1984). Immunological and biochemical properties of tonsillar lymphocytes. Acta Otolaryngol. (Stockholm) Suppl. **416**: 45–55.

Sugiyama M., Yamane H., Konishi K., and Kimura S. (1987). Subsets of tonsillar lymphocytes and activated cells in each subset analysed by two-color flow cytometry. Acta Otolaryngol. (Stockholm) **104**: 342–350.

Tohma S., and Lipsky P.E. (1991). Analysis of the mechanisms of T cell-dependent polyclonal activation of human B cell induction of human B cell responses by fixed activated T cells. J. Immunol. **146**: 2544–2552.

Van Den Eertwegh A.J.M., Nöelle R.J., Roy M., Shepherd D.M., Aruffa A., Ledbetter J.A., Boersma W.J., and Claassen E. (1993). In vivo CD40-gp 39 interactions are essential for thymus-dependent humoral immunity. I. In vivo expression of CD40 ligand, cytokines and antibody production delineates sites of cognate T–B cell interactions. J. Exp. Med. **178**: 1555–1565.

Velardi A., Grossi C.E., and Cooper M.D. (1985). A large subpopulation of lymphocytes with T helper phenotypes (Leu3/T4+) exhibits the property of binding to NK cell targets and granular lymphocyte morphology. J. Immunol. **134**: 8–64.

Velardi A., Mingari M.C., Moretta L., and Grossi C.E. (1986a). Functional analysis of cloned germinal center CD4[+] cells with natural killer cell-related features. Divergence from typical T helper cells. J. Immunol. **137**: 2808–2813.

Velardi A., Tilden A.B., Millo R., and Grossi C.E. (1986b). Isolation and charaterization of Leu7[+] germinal-center cells with the helper cell phenotype and granular lymphocyte morphology. J. Clin. Immunol. **6**: 205–215.

Whalen B.J., Tony H.P., and Parker D.C. (1988). Characterization of the effector mechanism of help for antigen-presenting and bystander resting B cell growth mediated by IA-restricted Th2 helper T cell lines. J. Immunol. **141**: 2230–2239.

